# Where Are All the Fish: Potential of Biogeographical Maps to Project Current and Future Distribution Patterns of Freshwater Species

**DOI:** 10.1371/journal.pone.0040530

**Published:** 2012-07-06

**Authors:** Danijela Markovic, Jörg Freyhof, Christian Wolter

**Affiliations:** Leibniz-Institute of Freshwater Ecology and Inland Fisheries, Berlin, Germany; University of Oxford, United Kingdom

## Abstract

The dendritic structure of river networks is commonly argued against use of species atlas data for modeling freshwater species distributions, but little has been done to test the potential of grid-based data in predictive species mapping. Using four different niche-based models and three different climate change projections for the middle of the 21st century merged pairwise as well as within a consensus modeling framework, we studied the variability in current and future distribution patterns of 38 freshwater fish species across Germany. We used grid-based (11×11 km) fish distribution maps and numerous climatic, topographic, hydromorphologic, and anthropogenic factors derived from environmental maps at a finer scale resolution (250 m–1 km). Apart from the explicit predictor selection, our modeling framework included uncertainty estimation for all phases of the modeling process. We found that the predictive performance of some niche-based models is excellent independent of the predictor data set used, emphasizing the importance of a well-grounded predictor selection process. Though important, climate was not a primary key factor for any of the studied fish species groups, in contrast to substrate preferences, hierarchical river structure, and topography. Generally, distribution ranges of cold-water and warm-water species are expected to change significantly in the future; however, the extent of changes is highly uncertain. Finally, we show that the mismatch between the current and future ranges of climatic variables of more than 90% is the most limiting factor regarding reliability of our future estimates. Our study highlighted the underestimated potential of grid cell information in biogeographical modeling of freshwater species and provides a comprehensive modeling framework for predictive mapping of species distributions and evaluation of the associated uncertainties.

## Introduction

Predicting future distribution patters of species following climate change projections becomes increasingly important in environmental management, species conservation and restoration planning at global, regional and local scales. Studies dealing with future species distribution patterns generally relay on niche-based species distributions models (SDMs) which relate the current conditions to the current species distributions and project these using climate change models (e.g., [1] [4]).

Recent studies on methodological aspects of SDMs have shown that application of consensus methods reduce model based uncertainty and increase reliability of the projections [1], [5] [–] [7]. In particular, Marmion et al. [5] have shown that consensus methods based on averaging of all methods provide robust projections and significantly increase the accuracy of species distribution forecasts. Additional important aspects of the predictive modeling process include a well substantiated predictor selection methodology and the investigation of the general predictability of future changes using the information on current conditions. Despite the overall high relevance of future projections, there is a general lack of studies comprising all these uncertainty aspects of the predictive modeling process.

Species distribution patterns are affected by a combination of environmental factors acting at different spatial and temporal scales [4], [8] [–] [10]. Climate is widely acknowledged as primary factor at the continental scale whilst topography, the land use and habitats become important at regional to local scales [11]. Because dendritic river network structures constrain species dispersal ability, describing spatial distribution patterns of freshwater species is inseparable from describing hierarchy, heterogeneity and lateral connectivity of river systems [12]. The dendritic river network structure is commonly argued against grid cell related data in biogeographical modeling of freshwater species such as fish (e.g. [2], [9]). However, most studies on fish distribution patterns are void of an explicit predictor selection process and combine detailed river and fish data at the site scale with the global environmental data (20 km grid cells) resulting in serious scale mismatches and biased data samples.

Freshwater biodiversity is particularly vulnerable to climate change not only because temperature is climate dependent, but also because other pressures on freshwater biodiversity such as human consumptions of ecological assets, nitrogen deposition and species invasions show increasing trends over recent decades [13]–[Bibr pone.0040530-Butchart1] [Bibr pone.0040530-Woodward1] [16]. Since the combined effect of climate and human stressors is likely to be amplified in the future compared to their individual effects, it is expected that there will be a considerable change in species composition and diversity loss [17].

Here, we faced the challenge of modeling distribution patterns of 38 freshwater fish species across Germany at the scale grain used for the national fish records (11 km×11 km grid cell). Grid cell related species distribution maps are common in presenting species occurrence information. Such data have already proven their potential to describe current and future distribution patterns of plant and terrestrial vertebrate species (e.g. [18], [19]). To our knowledge, the potential of fish occurrence maps in predictive biogeographical modeling at a comparable resolution has not been exploited. As the initial step we identified factors affecting species distributions. At the landscape to regional scale previous studies indicated the position along the upstream–downstream gradient, mean temperature and site elevation as major predictors of species distributions [2], [9], [20]. Across river basis at continental to global scale fish species distributions were commonly described by discharge, climate, topography, net primary productivity, dam characteristics, land use properties and population density [3], [8], [16]. In a fish species traits study based on literature data Goldstein and Meador [21] showed that fish distribution patterns vary according to stream size and substrate type. Consequently, we collected information on the substrate and stream size properties of each cell as well as information on climatic, hydromorphologic, topographic and anthropogenic properties.

The main objective of our study was the investigation of the potential of grid-cell data (atlas data) in biogeographical modeling of current and prediction of future distribution patterns of freshwater fish species. Our comprehensive modeling framework involved grid cell related fish and environmental data at a regional scale resolution, explicit predictor selection, different SDMs and different climate change projections. The potential of climate as a single factor describing current species distributions has been studied, as well as the importance of climate as a factor acting together with other properties of the studied cells. To ensure prediction reliability we restricted our analyses within the predictive modeling stage to only those SDMs considered in the validation process as good to excellent. We investigated variability in the estimated effects of climate change for the middle of the 21st century emerging from different stages of the modeling process for the consensus model and for all combinations of the different climate change models and the selected SDMs. Analysis of the variability in the predicted effects of climate change revealed important sources of uncertainty, some of which limit the possibility of providing reliable estimates of future changes in freshwater fish species distribution patterns.

## Methods

### Species distribution data

The German society for ichthyology (GFI, www.fishartenartlas.de) and the Federal Agency for Nature Conservation (BfN, www.bfn.de) have kindly provided actual fish species occurrence data in form of actual presence indicators for grid cells of the topographic map of Germany at scale of 1∶25 000 (TK25). Each TK25 cell covers an area of approximately 11×11 km size, forming a mesh of 2945 cells. The analyses have been reduced to 2935 cells due to missing environmental information for nine coastal cells. The grid cell based fish occurrence data are a result of merging presence/absence information from expert-verified surveys and catch reports of recreational and commercial fisheries across each of the German Federal States over the last two to three decades. We modeled only those fish species with sufficient reliable positive occurrences (more than 50), resulting in a set of 38 fish species (see Table 1).

**Table 1:** Fish species included in the analyses and their occurrence frequency.

Species code	Species name	Occurrence frequency
*Abrarama*	*Abramis brama*	0.385
*Albuatus*	*Alburnoides bipunctatus*	0.076
*Alburnus*	*Alburnus alburnus*	0.278
*Anguilla*	*Anguilla anguilla*	0.658
*Aspipius*	*Aspius aspius*	0.120
*Barbrbus*	*Barbus barbus*	0.279
*Barbtula*	*Barbatula barbatula*	0.482
*Blicrkna*	*Blicca bjoerkna*	0.283
*Caraelio*	*Carassius gibelio*	0.189
*Carasius*	*Carassius carassius*	0.274
*Chonasus*	*Chondrostoma nasus*	0.098
*Cobienia*	*Cobitis taenia*	0.183
*Cottobio*	*Cottus gobio*	0.420
*Cyprrpio*	*Cyprinus carpio*	0.406
*Esoxcius*	*Esox lucius*	0.523
*Gastatus*	*Gasterosteus aculeatus*	0.523
*Gobiobio*	*Gobio gobio*	0.616
*Gymnrnus*	*Gymnocephalus cernuua*	0.244
*Lampilis*	*Lampetra fluviatilis*	0.085
*Lampneri*	*Lampetra planeri*	0.337
*Leucatus*	*Leucaspius delineatus*	0.206
*Leucidus*	*Leuciscus idus*	0.181
*Leucscus*	*Leuciscus leuciscus*	0.384
*Lotalota*	*Lota lota*	0.165
*Misgilis*	*Misgurnus fossilis*	0.145
*Percilis*	*Perca fluviatilis*	0.576
*Phoxinus*	*Phoxinus phoxinus*	0.248
*Pungtius*	*Pungitius pungitius*	0.253
*Rhodarus*	*Rhodeus amarus*	0.159
*Rutiilus*	*Rutilus rutilus*	0.648
*Salmutta*	*Salmo trutta*	0.597
*Salvalis*	*Salvelinus fontinalis*	0.104
*Sanderca*	*Sander lucioperca*	0.248
*Scarlmus*	*Scardinius erythrophthalmus*	0.334
*Siluanis*	*Silurus glanis*	0.124
*Squaalus*	*Squalius cephalus*	0.447
*Thymllus*	*Thymallus thymallus*	0.319
*Tincinca*	*Tinca tinca*	0.481

### Environmental data

Baseline data. The baseline data consist of approximately 50 climatic, hydromorphologic, topographic and anthropogenic predictors considered potentially relevant for general physiological and behavioral properties of studied species. To minimize the effect of spatial redundancy in the environmental information, we used only those data sets with scale grains ≤1.1 km, i.e., at least ten times smaller than the scale grain of our study. For calculation of the TK25 cell specific values we used ArcView, version 3.2 (ESRI).

Climate related predictors were based on the 30 arc-seconds resolution (approx. 1 km) of the WorldClim (www.worldclim.org) data set. The WorldClim data set consist of 19 bioclimatic predictors, monthly mean, minimum and maximum temperature and monthly total precipitation derived from the station time series data for the second half of the 20th century [22]. The bioclimatic predictors summarize the effects of annual trends, seasonality and potentially limiting environmental factors on the species distributions. Actual evapotranspiration is estimated using the Turc's formula [23] while the cell runoff resulted from the general water balance equation in which the groundwater storage is neglected due to the fact that the calculation period is several decades. Temperature averages for March–May and May–July were added to the climate related predictor data set to account for pronounced temperature requirements of fish during reproduction and early development stages.

The initial set of hydromorphological predictor variables considered the following TK25 cell indicators: maximum cumulative length of the upstream flow network, maximum and minimum basin area, dominant river type category and the maximum Strahler order. All listed indicators except the river type category were based on the CCM River and Catchment Database, version 2.1 (CCM2, [24]). Predictors related to basin area and upstream flow network length indirectly reflect basin heterogeneity and thus potential species diversity. The river type was derived from the German national typology classifying all flowing waters according to their morphological, geological, hydrological and biological properties into 24 river types [25]. For the purposes of our study, the categories were merged with focus on the major substrate type into following classes: (1) alpine streams; (2) boulder and gravel dominated large streams in the alpine foothills; (3) fine to coarse substrate dominated siliceous highland streams; (4) fine to coarse substrate dominated calcareous highland streams; (5) gravel dominated streams; (6) sand dominated streams; (7) organic substrate dominated streams; (8) loess and loam dominated streams; (9) marshland streams of the coastal plains; (10) small streams in riverine floodplains without dominant substrate type.

Germany has a pronounced North-South altitudinal gradient, with Alps in the South and the North- and the Baltic Sea in the North and North-East, respectively, wherefore the topographical predictor set included the average TK25 cell altitude as well as the mean and maximum slope as surrogates for the water velocity at a resolution of 250 m.

The anthropogenic predictor set included the population density data and the Corine Land Cover data (CLC) of the European Environment Agency (www.eea.europa.eu) at a resolution of 250 m. The initial 44 land use types were summarized into following ecologically meaningful land use categories: urban/commercial, agriculture, forest, grassland, freshwater, marine and other. For each TK25 cell dominant land use type as well as TK25 cell surface percentages covered by each category were estimated.

#### Future climate predictions

For evaluation of the potential future species distributions we used the downscaled climate projections for the middle of the 21st century (2040–2069, referred further as 2050s) from the CIAT data portal (www.ccafs-climate.org) at 30 arc-seconds spatial resolution. The CIAT data set is based on the general circulation models (GCMs) from the IPCC Fourth Assessment Report. Since the statistical downscaling framework is fixed to the baseline climate as defined by the WorldClim data set, consistency between our “baseline” and “future” climate datasets is assured. The most commonly used in the ecological studies are the projections of the HadCM3 model [26] for the IPCC's A2 and B1 storylines developed at the Hadley Centre for Climate Prediction (e.g. [1], [2], [4]. A2 storyline describes a very heterogeneous world with continuously increasing global population and regionally oriented economic growth [27]. B1 storyline describes a world characterized with global population that peaks in mid-century and declines thereafter, and introduction of clean and resource-efficient technologies [27]. As energy requirements are highest for the scenarios from the A1 family, we selected the A1b scenario of a world with continuously increasing global population, very rapid economic growth and maximum energy requirements that are balanced across all energy sources. In addition to HadCM3 we used projections of the ECHAM5 [26] model (Max Planck Institute for Meteorology, Germany) and the IPSL-CM4 [26] model (Institute Pierre Simon Laplace, France).

### Analysis framework

Statistical analyses and model building were carried out using various own MATLAB (version 6.0.0.88) and R (version 2.14) codes combined with the BIOMOD library [28] (version 1.1–5) and the ME package [29] (version 3.3.3a).

#### Predictor variables selection

In order to avoid multicollinearity, for each of the predictor categories (climate, hydromorphology, topography and anthropogenic influences) using the principal component analysis (PCA) and the univariate analysis we selected a representative predictor set which satisfied the condition of pairwise correlations below 0.75. Further reduction of the predictor number was an iterative process supported with the univariate analysis of the relevance of each individual predictor and analysis of the predictor relevance within the multivariate model setup. Thereby, we used three selection criteria: (1) average univariate area under the receiver operating characteristic ≥0.65; (2) average permutation importance ≥5%; (3) at least for half of the considered species the respective parameter should be selected by the SDM's. If at least two out of these three criteria were fulfilled, the predictor was considered as a relevant factor affecting fish species distribution across the studied area at the studied scale.

#### Modeling current fish species distributions

Species distribution patterns across Germany were modeled using Generalised Linear Models (GLM), Generalised Additive Models (GAM), Random Forest (RF) and the Maximum Entropy Approach (ME). GLM is one of the best established techniques [10] and is an extension of the linear models that deal with the non-normal error distributions. GAM is a non-parametric extension of GLM widely used for analyses and description of the distribution patters of fish species (e.g. [7], [30]). Here, cubic spline smoothers with four degrees of freedom were used for fitting the GAM models. RF is a classification method which creates a certain number of trees (here 500) constructed using a different bootstrap sample from the original data [31]. This technique has shown to have a good ability to predict observed fish species distributions. In the ensemble modeling experiment by Grenouillet et al. [7] among eight tested statistical methods, RF came closest to the average model. Unlike the GLM, GAM and RF that use species presence and absence data, ME uses presence data only. Each site is assigned a probability value whereas the probability distribution itself is constrained by the properties of the original data [29]. Among all probability distributions which satisfy the given constraints, ME chooses the one with the maximum entropy.

For all SDMs except the ME, searching for a parsimonious model involved analyses of the model improvement based on the Akaike Information Criterion (AIC) through stepwise adding of new variables and leaving those out which do not significantly improve the fit. Estimates of the variable contributions in the individual models were based on the normalized correlations between the predictions using the original data and the predictions using the data in which the variable of interest is randomly permutated. The modeling process involved a random splitting of the data samples into the calibration (70%) and the validation data set (30%) repeated 10 times for each SDM. Species occurrence probabilities were transformed to the presence/absence information using the SDM specific thresholds which maximize both, sensitivity (the true positive rate) and the specificity (the true negative rate). The modeled presence/absence results were merged to a single model within a consensus modeling framework involving averaging across all SDMs including their replications (see [5]). Within the averaging procedure, a particular cell is assigned the presence indicator “1” only if the same indicator was estimated for more than 50% of the considered models. Agreement between the observed and modeled species distribution patterns was quantified by sensitivity, specificity and the coincidence rate. The later describes the ratio of the number of “correctly” predicted cells (presences and absences) to the total number of cells. General performance of the calibrated models was classified using the area under the receiver operating characteristic curve (ROC score) [e.g. 30]: 0.5–0.6 (“fail”), 0.6–0.7 (“poor”), 0.7–0.8 (“fair”), 0.8–0.9 (“good”), 0.9–1 (“excellent”).

#### Patterns in the species' environmental responses

Identification of species groups according to their responses to different environmental predictors was performed using Fuzzy c-mean clustering technique (see [32]). Environmental responses were calculated as a ratio of the group based sum of predictor importance estimates and the sum of all importance estimates. In order to compare the variation of the environmental responses across different SDMs the clustering was applied to the results of each considered technique separately.

#### Future species distributions and associated uncertainties

For the species identified in the previous step as climate sensitive, potential future distribution maps for the period 2040–2069 under A1b scenario have been created according to different climate models and the SDMs identified as suitable for modeling fish species distribution at the particular scale resolution. A joint future consensus pattern was estimated through averaging of all pairwise combinations of SDMs (including repetitions) and climate models. Within the averaging procedure, species are considered to occur in a given cell if more than 50% of the models predicted species occurrence for the particular cell. The scope of our study involves not only derivation of future fish species distribution patterns, but also estimation of uncertainties and projections variability associated with the predictor variable selection, SDM, selected future climate projection and a relationship between the predictor ranges for the baseline and the future situation. We studied differences in the spatial distribution patterns of selected fish species in terms of distribution coincidence rates, differences in the estimated “habitat” losses and gains as well as percentages of study area where the future projection is unreliable due to mismatches between the predictor calibration and future range. Thereby, the “loss” (“gain”) is defined as the ratio of grid cell number where the species is currently present (absent) but predicted to be absent (present) in the future to the total number of currently occupied cells. The coincidence rate between e.g. projections of two different climate models correspond to the ratio of coinciding presence and absence cells for these two models and the total number of TK25 cells.

## Results

### Predictor variables selection

**Figure 1:**
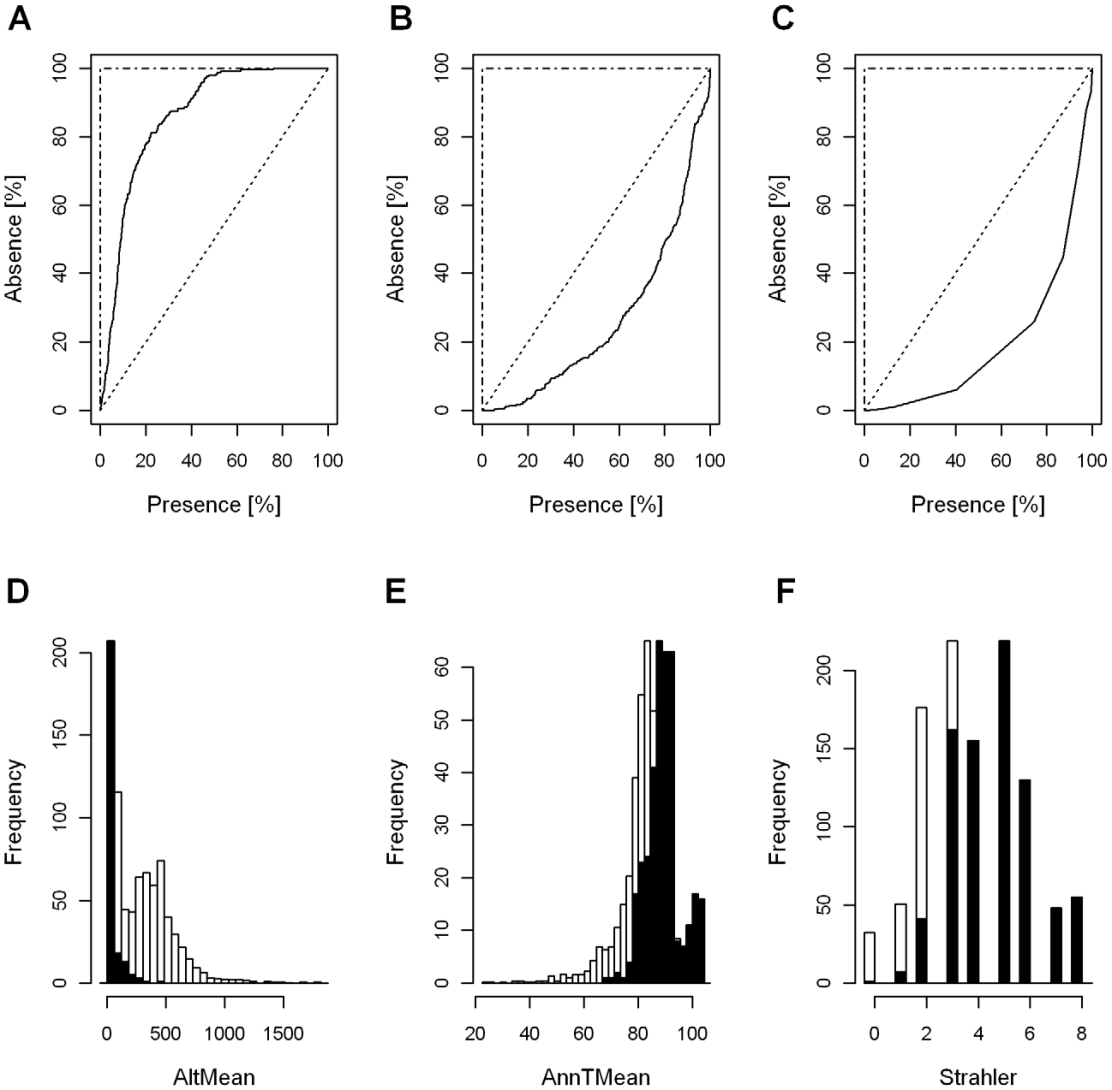
Lorenz Curve and the empirical probability distributions. (A, D) *L. fluviatilis*, (B, E) *S. glanis* (b, e) and (C, F) *B. barbus*. The ROC score of 0.85 for AltMean (*L. fluviatilis*), 0.73 for AnnTMean (*S. glanis*) and 0.8 for Strahler (*B. barbus*) indicate high discriminatory power of the individual predictors in describing species presence (shaded bars) and absence patterns.

From the baseline data set consisting of approximately 50 environmental predictors, through synthesis of the PCA, univariate and the correlation analysis we selected the major factors representing the climatic, hydromorphologic, topographic and anthropogenic properties of the studied grid cells (see Table 2 and Figure S1). Exemplarily, the first two PCA's of the precipitation related data set describing 74% and 24% of the joint variability were correlated most with annual mean precipitation (AnnPMean) and the precipitation seasonality (PSeason). Consequently, only these two precipitation related predictors were considered for further analysis. Also, due to high correlations with the mean annual temperature (AnnTMean) and AnnPMean, both evapotraspiration and runoff had to be excluded from the analysis scope. Within the subsequent analysis step we quantified the overall performance of the selected factors in describing spatial distribution patterns of the selected fish species and performed the final predictor selection. The results summarized in Table 2 indicate that mean temperature of wettest (TWetQuart) and driest quarter (TDryQuart) have lowest predictive power compared to other climatic factors and, also, that none of the considered anthropogenic factors hold significant predictive power. The univariate regression has additionally enabled us to identify the key factors of species's distributions. In particular, as shown in Figure 1, the mean altitude (AltMean) describes the spatial patterns of Lampetra fluviatilis (ROC score of 0.85), AnnTMean is the dominant factor in describing the spatial pattern for Silurus glanis (ROC score of 0.73) and habitat selection of Barbus barbus can be described using the Strahler stream order (ROC score of 0.8).

**Table 2:** Environmental predictors and their discriminatory power.

Predictor	Description	ROC score	PI	No
AnnTMean	Annual mean temperature (°C)	0.66	7.6	26
DiuTRange	Mean diurnal temperature range (°C)	0.58	8.1	23
Isotherm	Isothermality (-)	0.58	7.7	21
TSeason	Mean temperature seasonality (°C^2^)	0.58	9.9	23
Tmax	Maximum temperature of warmest month (°C)	0.61	3.0	18
TWetQuar	Mean temperature of wettest quarter (°C)	0.66	1.8	18
TDryQuar	Mean temperature of driest quarter (°C)	0.55	2.6	20
AnnPMean	Annual mean precipitation (mm)	0.65	6.1	29
PSeason	Mean precipitation seasonality (-)	0.56	3.6	19
AltMean	Mean altitude (m)	0.66	17.8	27
CumLenkm	Maximum cumulative length of the upstream flow network (km)	0.65	3.9	24
Strahler	Maximum Strahler order	0.64	12.9	36
RtypMost	Dominant river type	0.61	11.3	32
Lusemax	Dominant land use type	0.55	1.7	26
Popmean	Mean population density (Inhabitants/km^2^)	0.57	2.0	24

“ROC score” is the mean ROC score based on the univariate analysis; “PI” is the mean permutation importance of each individual predictor for GLM, GAM, RF and ME based multivariate SDMs; “No” is the number of species for which the respective predictor variable was identified as statistically significant (the total number of considered species is 38).

Finally, the “basic set of predictors” affecting spatial distribution of studied fish species entails the following characteristics of the studied grid cells (see Table 2 for abbreviation explanations): climatic (AnnTMean, Isotherm, TSeason and AnnPMean), topographic (AltMean) and hydromorphologic (CumLenkm, Strahler and RtypMost).

### Modeling current fish species distributions

**Figure 2:**
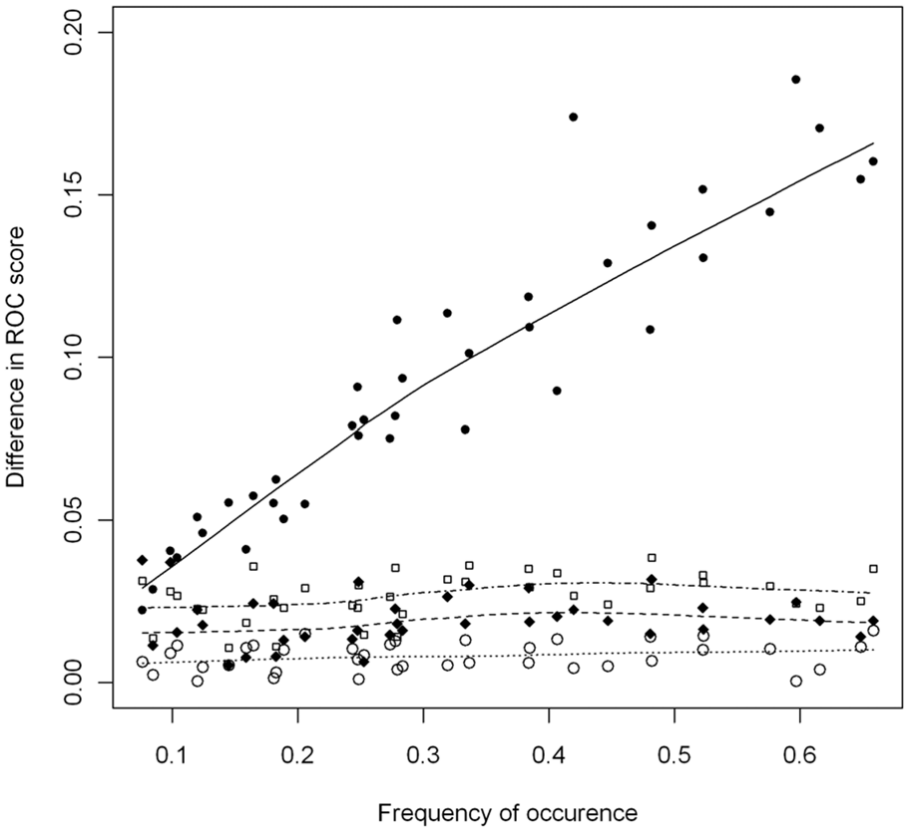
Relationship between species occurrence frequency and the ROC score. The ROC score difference is calculated as the absolute deviation of the average validation ROC score for the particular method from the total average validation ROC score for all applied SDMs (GLM, GAM and RF). The trend line is based on locally weighted scatterplot smoothing: ME (solid line), GAM (dotted line), GLM (dashed line), RF (dot-dash line).

The model calibration process was initiated using the above described “basic set of predictors”. Due to the stepwise predictor elimination in a search for the most parsimonious model, the final number of model predictors was commonly between four and six. Summary of the average statistical performance of the calibrated models per individual fish species is given in Table 3. The calibration ROC score for the RF based SDMs indicated “excellent” models (ROC score>0.9) for all 38 species, but the average validation score of 0.84 emphasized that there is a considerable performance drop when it comes to the validation sample. For GAM and GLM based SDMs, both average calibration and validation scores were around 0.8. ME had lowest average calibration and validation score (0.76 and 0.72, respectively) and, additionally, ME's performance has shown to be dependent on species's occurrence frequency. Namely, deviation of the ME based ROC validation score from the average validation ROC score (calculated as an average per species for all methods and all repetitions) was positively correlated with the occurrence frequency (Figure 2). For all SDMs applied, standard deviations of the ROC score estimates were ≤0.02 whereas sensitivities and specificities were >90 for RF and between 70 and 80 for all other SDMs (Table 3). Within the applied modeling approach both, percentage of presence and absence correctly predicted were simultaneously maximized, resulting in equal specificity and sensitivity estimates. Because our intention was to use the model results to investigate the potential influence of future changes, to align with assumption that species niches are stable over time, the model representations of those niches have to be robust and stable. Due to above described performance pitfalls, ME was considered inappropriate for the predictive modeling of the fish species distributions at the particular scale resolution and was not included in the consensus model building. The average performance of the consensus models (based on GAM, GLM and RF and their repetitions) is 0.83 (“good”, see Table 3). To ensure robustness and reliability of our forecast, only those species with average GAM, GLM and RF based validation ROC score ≥0.8 (Table 3) were analyzed within the subsequent steps.

**Table 3:** Summary of the model performance for the current conditions.

	Calibration ROC score				Validation ROC score				Sensitivity & Specificity				ROC
Species code	GAM	GLM	RF	ME	GAM	GLM	RF	ME	GAM	GLM	RF	ME	CM
*Abrarama*	0.79	0.78	0.97	0.72	0.78	0.77	0.82	0.68	71	70	92	69	0.82
*Albuatus*	0.90	0.85	0.98	0.90	0.88	0.83	0.90	0.85	81	76	93	86	0.85
*Alburnus*	0.83	0.81	0.98	0.78	0.81	0.80	0.86	0.74	75	74	93	75	0.84
*Anguilla*	0.74	0.73	0.97	0.62	0.73	0.73	0.78	0.58	67	67	91	61	0.80
*Aspipius*	0.92	0.90	0.99	0.89	0.91	0.88	0.93	0.86	84	82	95	86	0.89
*Barbrbus*	0.90	0.88	0.98	0.80	0.89	0.87	0.90	0.77	83	80	95	76	0.88
*Barbtula*	0.78	0.75	0.97	0.69	0.77	0.75	0.82	0.64	70	68	92	67	0.80
*Blicrkna*	0.81	0.80	0.97	0.76	0.81	0.80	0.84	0.72	73	71	92	73	0.83
*Caraelio*	0.78	0.77	0.97	0.76	0.77	0.76	0.80	0.72	72	71	90	73	0.83
*Carasius*	0.76	0.75	0.97	0.72	0.75	0.75	0.79	0.69	68	68	91	69	0.81
*Chonasus*	0.92	0.87	0.99	0.89	0.90	0.85	0.92	0.85	84	78	94	88	0.88
*Cobienia*	0.91	0.90	0.98	0.85	0.89	0.88	0.90	0.83	83	82	94	81	0.89
*Cottobio*	0.88	0.86	0.98	0.74	0.88	0.86	0.91	0.71	80	78	95	70	0.86
*Cyprrpio*	0.74	0.73	0.96	0.68	0.72	0.71	0.77	0.64	68	67	91	66	0.79
*Esoxcius*	0.77	0.75	0.97	0.66	0.74	0.74	0.79	0.62	70	69	91	64	0.81
*Gastatus*	0.79	0.77	0.97	0.68	0.78	0.77	0.83	0.64	72	71	92	66	0.82
*Gobiobio*	0.78	0.76	0.97	0.64	0.76	0.75	0.79	0.60	70	69	92	63	0.80
*Gymnrnus*	0.84	0.84	0.97	0.80	0.83	0.83	0.87	0.76	76	76	93	76	0.85
*Lampilis*	0.92	0.90	0.98	0.91	0.90	0.89	0.92	0.88	85	82	94	88	0.89
*Lampneri*	0.79	0.76	0.97	0.72	0.77	0.75	0.82	0.68	72	70	92	69	0.82
*Leucatus*	0.76	0.75	0.96	0.77	0.74	0.74	0.78	0.70	70	69	91	75	0.81
*Leucidus*	0.85	0.81	0.97	0.83	0.83	0.81	0.86	0.78	76	75	92	79	0.84
*Leucscus*	0.79	0.76	0.97	0.70	0.78	0.75	0.82	0.66	72	68	92	68	0.81
*Lotalota*	0.83	0.81	0.97	0.82	0.82	0.80	0.86	0.77	75	74	92	80	0.84
*Misgilis*	0.88	0.87	0.98	0.84	0.86	0.86	0.87	0.81	80	79	93	80	0.87
*Percilis*	0.76	0.75	0.97	0.65	0.75	0.74	0.79	0.61	70	68	92	63	0.81
*Phoxinus*	0.83	0.79	0.98	0.78	0.81	0.78	0.84	0.74	74	71	92	75	0.83
*Pungtius*	0.89	0.89	0.98	0.83	0.88	0.88	0.90	0.81	82	81	95	79	0.89
*Rhodarus*	0.81	0.80	0.97	0.78	0.78	0.78	0.81	0.75	74	73	91	73	0.84
*Rutiilus*	0.75	0.74	0.96	0.63	0.73	0.73	0.77	0.59	68	67	91	62	0.79
*Salmutta*	0.84	0.81	0.97	0.67	0.83	0.80	0.85	0.64	77	73	93	63	0.84
*Salvalis*	0.79	0.77	0.97	0.80	0.76	0.76	0.80	0.74	71	69	90	79	0.80
*Sanderca*	0.82	0.81	0.97	0.78	0.82	0.81	0.85	0.74	74	73	93	76	0.83
*Scarlmus*	0.75	0.74	0.96	0.71	0.73	0.72	0.77	0.66	67	66	91	69	0.80
*Siluanis*	0.88	0.86	0.98	0.86	0.86	0.85	0.89	0.82	79	77	93	84	0.86
*Squaalus*	0.81	0.78	0.97	0.70	0.79	0.77	0.82	0.66	73	70	92	67	0.82
*Thymllus*	0.84	0.81	0.98	0.76	0.83	0.81	0.86	0.72	76	73	93	72	0.83
*Tincinca*	0.73	0.72	0.96	0.66	0.71	0.71	0.76	0.62	66	65	91	64	0.79
**Mean**	**0.82**	**0.80**	**0.97**	**0.76**	**0.80**	**0.79**	**0.84**	**0.72**	**74**	**73**	**92**	**73**	**0.83**

The table values indicate averages over all model repetitions. Standard deviation of the ROC score estimates for the calibration and the validation data sets is less than 0.01, 0.02, respectively, for all methods. “ROC CM” is the ROC score of the consensus models.

In order to identify which fish species out of those selected in the previous step are particularly sensitive to climate, we additionally ran all models using only climatic predictors. Again, RF based average ROC score was highest for both, calibration and the validation data set (0.97 and 0.80 respectively) whereas GAM and GLM had a similar performance with ROC scores around 0.75. Interestingly the average validation score based on GAM, GLM and RF for Aspius aspius, Chondrostoma nasus, Cobitis taenia, Cottus gobio, L. fluviatilis, Misgurnus fossilis, Pungitius pungitius and S. glanis was ≥0.8 suggesting that climate is an important factor that affects spatial distributions of these species. However, a comparison between the model performances using all considered factors (Table 3) and using climatologic factors only (Table 4) reveals a significant performance drop for the latter, except for C. gobio and S. glanis, which appeared as particularly climate sensitive.

**Table 4:** Summary of GAM, GLM and RF models based on AnnTMean, Isotherm, TSeason and AnnPMean.

	Validation ROC score			ROC score change			Sensitivity & Specificity		
Species code	GAM	GLM	RF	GAM	GLM	RF	GAM	GLM	RF
*Albuatus*	0.78	0.74	0.86	−0.09	−0.08	0.07	71	68	91
*Alburnus*	0.76	0.76	0.81	−0.04	−0.04	0.11	69	69	91
***Aspipius***	0.81	0.79	0.87	−0.1	−0.09	0.05	73	71	92
*Barbrbus*	0.75	0.74	0.81	−0.13	−0.12	0.07	71	68	92
*Blicrkna*	0.76	0.74	0.80	−0.04	−0.05	0.13	69	67	91
***Chonasus***	0.82	0.78	0.86	−0.07	−0.07	0.05	75	72	91
***Cobienia***	0.81	0.74	0.88	−0.08	−0.15	0.08	74	66	93
***Cottobio***	0.83	0.78	0.88	−0.05	−0.08	0.07	74	70	94
*Gymnrnus*	0.79	0.77	0.82	−0.03	−0.06	0.1	71	69	92
***Lampilis***	0.82	0.76	0.85	−0.07	−0.13	0.05	75	67	91
*Leucidus*	0.73	0.68	0.80	−0.09	−0.13	0.11	68	61	91
*Lotalota*	0.72	0.69	0.80	−0.09	−0.11	0.1	66	65	90
***Misgilis***	0.80	0.76	0.84	−0.05	−0.09	0.1	73	70	91
*Phoxinus*	0.72	0.65	0.80	−0.08	−0.13	0.13	67	60	91
***Pungtius***	0.81	0.78	0.86	−0.06	−0.09	0.08	75	71	93
*Salmutta*	0.78	0.71	0.82	−0.05	−0.09	0.12	71	66	93
*Sanderca*	0.77	0.75	0.81	−0.04	−0.05	0.12	70	70	91
***Siluanis***	0.83	0.81	0.85	−0.03	−0.04	0.08	75	73	92
*Thymllus*	0.76	0.72	0.82	−0.07	−0.09	0.11	70	66	92
**Mean**	**0.75**	**0.72**	**0.80**	**−0.07**	**−0.09**	**0.09**	**69**	**67**	**91**

ROC score, sensitivities and specificities indicate averages over all model repetitions. Standard deviation of the ROC score estimates for the validation data sets ranges from 0.01 to 0.03 for GLM and GAM based models and from 0.01 to 0.02 for RF. The “ROC score change” is the difference between the mean ROC for the models based on climatic factors and the mean ROC for models considering climatic, topographic and hydromorphologic predictors (see Table 3).

**Figure 3:**
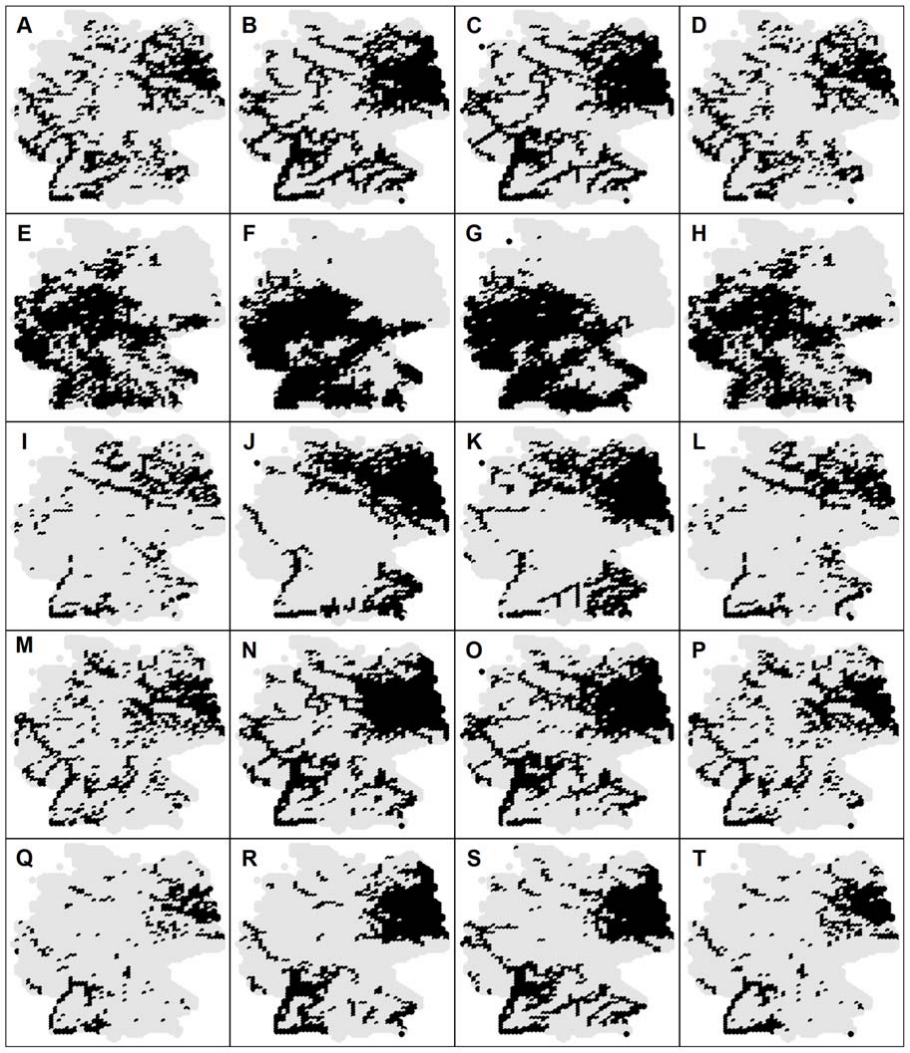
Current species distributions and their model representations. Each row shows species specific results: (A–D) *A. alburnus*, (E–H) *C. gobio*, (I–L) *L. lota*, (M–P) *S. lucioperca* and (Q–T) *S. glanis*. The first column indicates current species distribution and is followed by the model representations using GAM, GLM and RF, respectively. Gray dots indicate centroids of the grid cells across the whole study area (Germany).

Figure 3 shows the current species distribution patterns and their GAM, GLM and RF model representations for Alburnus alburnus, C. gobio, Lota lota, Sander lucioperca and S. glanis. Sensitivies and specificities were highest for the RF based models (Table 3 and Figure 3) implying that for this particular SDM, the modeled species distribution patterns are closest to the observed ones. The average difference between the observed and the estimated species' occurrence frequency was highest for GLM (+0.12), slightly lower for GAM (+0.11) and lowest for RF (+0.02), but all methods agreed that the currently occupied habitat is probably smaller that the potentially suitable habitat. Pairwise comparison of the model outputs revealed that GAM and GLM have a high agreement in terms of the average coincidence rate (93±1%) whereas between GAM and RF and between GLM and RF, the agreement was considerably lower (79±3% and 77±2%, respectively). Consequently, for the species distribution patterns shown in Figure 3 the average sensitivity and the specificity of the consensus model was lower (80±2%) than that of the RF model (98±1%) but higher than that of the GLM and GAM (76±2%).

### Patterns in the species' environmental responses

Four distinct fish species groups have been identified according to their responses to climatic, hydromorphologic and topographic factors using the Fuzzy c-means clustering (see Table 5). Group 1 is mainly determined by altitude (e.g. P. pungtius, C. taenia, L. fluviatilis), group 2 by the hydromorphologic factors (e.g. Salmo Trutta, Phoxinus phoxinus, Thymallus thymallus), group 3 by a combination of hydromorphologic and climatic factors (e.g. S. lucioperca, S. glanis, A. alburnus, C. gobio), and group 4 by a rather equal contribution of all factors (e.g. C. nasus, A. aspipius, Gymnocephalus cernuua). Surprisingly, there was no group of species primarily determined by climatic factors.

**Table 5:** Mean permutation importance of the predictor groups, cluster id's and the corresponding cluster membership grades.

	GAM					GLM					RF				
Species code	C	H	HY	Fc	MG	C	H	HY	Fc	MG	C	H	HY	Fc	MG
*Albuatus*	0.5	0.4	0.1	2	0.5	0.3	0.3	0.4	3	1.0	0.4	0.3	0.2	3	0.8
*Alburnus*	0.4	0.1	0.5	3	1.0	0.4	0.0	0.6	1	0.6	0.5	0.1	0.4	4	0.4
*Aspipius*	0.3	0.3	0.4	1	0.9	0.3	0.3	0.4	3	1.0	0.3	0.2	0.4	2	0.9
*Barbrbus*	0.2	0.3	0.5	1	0.8	0.3	0.1	0.6	1	0.9	0.2	0.2	0.6	2	0.5
*Blicrkna*	0.4	0.2	0.4	3	0.4	0.3	0.1	0.6	1	0.7	0.5	0.2	0.3	2	0.4
*Chonasus*	0.4	0.3	0.3	1	0.6	0.4	0.2	0.4	3	1.0	0.4	0.2	0.4	2	0.8
*Cobienia*	0.2	0.6	0.2	2	0.9	0.2	0.6	0.2	4	1.0	0.2	0.6	0.2	1	0.9
*Cottobio*	0.5	0.1	0.4	3	0.8	0.3	0.0	0.7	1	0.9	0.5	0.2	0.2	4	0.6
*Gymnrnus*	0.2	0.4	0.4	1	0.8	0.1	0.3	0.5	3	0.5	0.4	0.3	0.3	3	0.7
*Lampilis*	0.3	0.5	0.2	2	0.9	0.1	0.5	0.3	4	0.7	0.2	0.6	0.2	1	1.0
*Leucidus*	0.2	0.4	0.4	1	0.6	0.2	0.5	0.3	4	0.7	0.3	0.4	0.3	1	0.4
*Lotalota*	0.3	0.3	0.4	1	0.9	0.2	0.1	0.7	1	0.6	0.4	0.3	0.3	3	1.0
*Misgilis*	0.3	0.5	0.2	2	0.9	0.3	0.5	0.2	4	0.9	0.3	0.3	0.3	3	0.8
*Phoxinus*	0.2	0.2	0.6	4	0.8	0.1	0.0	0.9	2	1.0	0.4	0.3	0.3	3	1.0
*Pungtius*	0.1	0.8	0.1	2	0.7	0.1	0.8	0.1	4	0.9	0.3	0.6	0.1	1	0.9
*Salmutta*	0.3	0.1	0.6	4	0.9	0.1	0.0	0.9	2	1.0	0.5	0.2	0.3	4	0.4
*Sanderca*	0.4	0.0	0.6	3	0.8	0.4	0.0	0.6	1	0.9	0.6	0.1	0.4	4	0.9
*Siluanis*	0.5	0.0	0.5	3	0.9	0.5	0.1	0.5	1	0.6	0.7	0.1	0.2	4	0.8
*Thymllus*	0.3	0.1	0.7	4	0.9	0.0	0.0	1.0	2	0.9	0.4	0.2	0.4	2	1.0
**Mean**	0.3	0.3	0.4	-	-	0.2	0.2	0.5	-	-	0.4	0.3	0.3	-	-

Table values indicate average permutation importance in percent of each predictor group: climate (C), altitude (H) and hydromorphology (HY). Members of the C group are AnnTMean, Isotherm, TSeason and AnnPMean, in the HY group are Strahler, CumLenkm and RtypMost while H is based on the permutation importance of AltMean. Degree to which each species belong to a Fuzzy cluster Fc is indicated by the membership grade MG.

### Future species distributions and associated uncertainties

The average annual temperature across the studied TK25 grid cells for 2050s under A1b is according to the ECHAM5 model slightly lower (10.8°C) than the projections for the HadCM3 and IPSL-CM4 models (11.1°C, see Table 6) which is however, 2.4°C higher than AnnTMean of the second half of the 20th century (8.4°C). Regarding Isotherm, all climate models predict similar values and the variation is in almost the same range as that for the 20th century climate. The ECHAM5 model, unlike the other two models, predicts lower TSeason than that of the 20th century. AnnPMean was predicted to decrease by a similar amount for all climate models. Although PSeason and Tmax were shown to be not significant overall, they might have an effect for some of the selected species. Therefore, we added these to the “basic set of predictors” within this part of our analyses. Tmax was predicted to increase by 2.8°C (ECHAM5) to 4.1°C (HadCM3), while PSeason has lower (ECHAM5 and HadCM3) as well higher (IPSL-CM4) values then the average 20th century value for the study region.

**Table 6:** Major characteristics of the bioclimatic predictor variables for the 20th century climate and for the future climate projections (2050s).

Predictor	Unit	Mean and range 20th century Worldclim	Mean and range for 2050s ECHAM5	Mean and range for 2050s HADCM3	Mean and range for 2050s IPSL-CM4
AnnTMean	°C	8.4 (2.5–10.4)	10.8 (4.8–12.9)	11.1 (5.1–13.2)	11.1 (5.0–13.1)
Isotherm	°C	3.1 (2.3–3.5)	3.2 (2.4–3.5)	3.1 (2.3–3.5)	3.1 (2.2–3.5)
TSeason	°C^2^	63.7 (54.5–76.8)	62.7 (52.7–73.6)	66.8 (58.2–79.2)	65.3 (57.4–77.8)
AnnPMean	mm	732.1 (482.4–1414.1)	716.3 (473.3–1323.1)	715.3 (467.0–1363.6)	713.4 (473.6–1374.4)
*Tmax*	°*C*	*22.7 (19.4–25.5)*	*25.5 (21.3–28.7)*	*26.8 (22.4–30.1)*	*25.9 (22.2–28.6)*
*PSeason*	*-*	*20.7 (9.5–37.1)*	*15.0 (9.9–27.4)*	*17.2 (11.0–26.9)*	*22.5 (12.3–36.6)*

See Table� 2 for abbreviation explanations.

**Figure 4:**
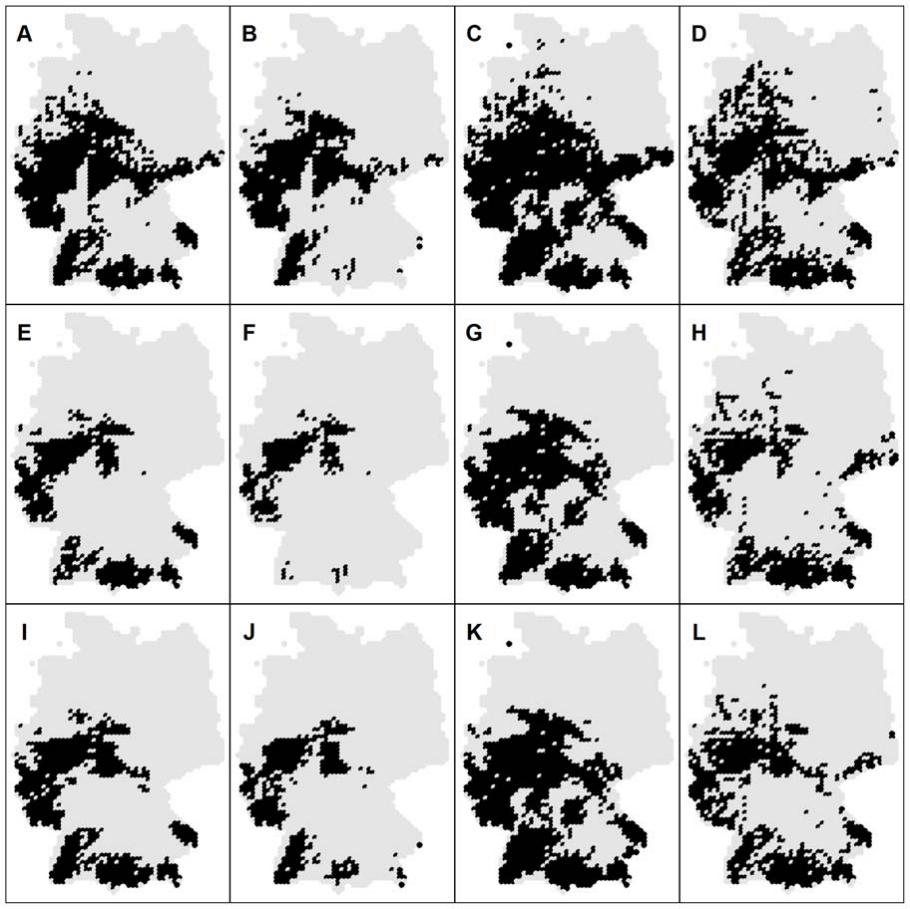
Potential distribution patterns for 2050s under A1b scenario for *C. gobio*. Each row shows the results of the particular climate model: (A–D) ECHAM5, (E–H) HADCM3 and (I–L) IPSL-CM4. The first column shows the result of the mean consensus model based on all repetitions of GAM, GLM and RF, followed by the mean future projections using all repetitions of the individual models: GAM, GLM and RF, respectively. Gray dots indicate centroids of the grid cells across the whole study area (Germany).

**Figure 5:**
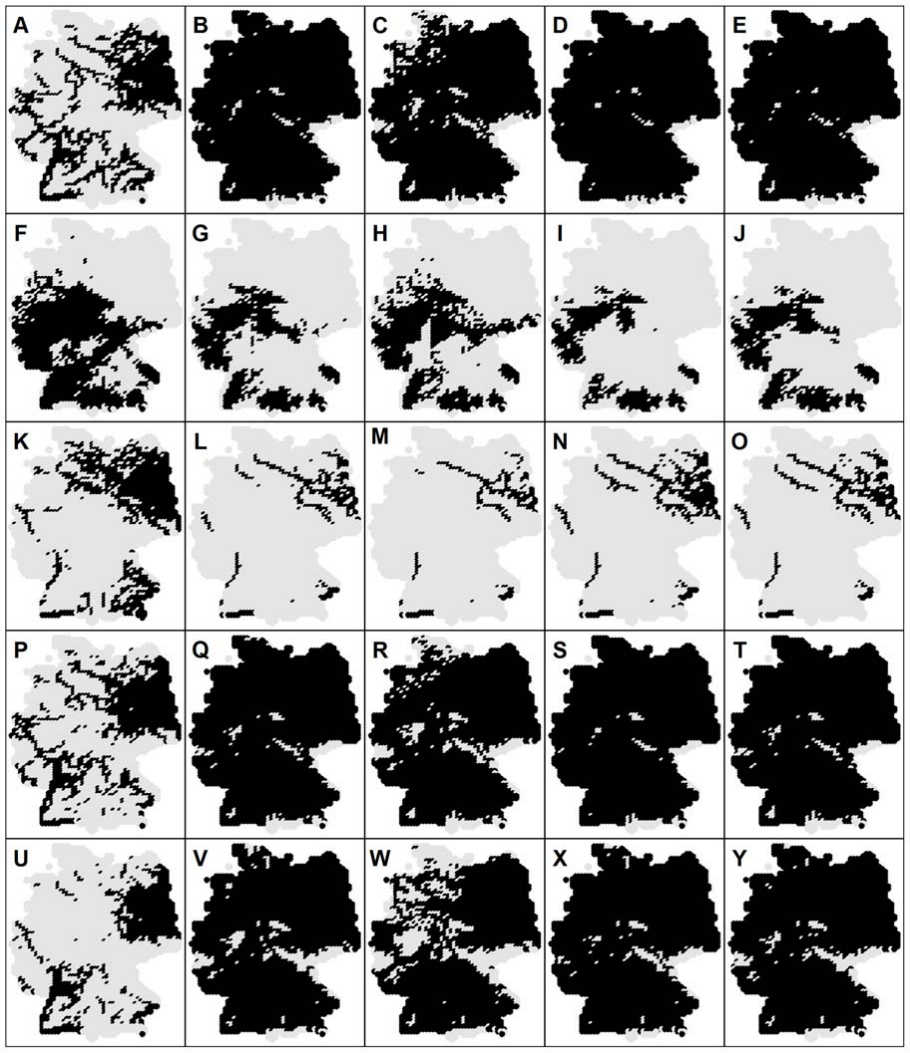
Current and the potential future distribution patterns of selected species for 2050s under A1b scenario. Each row shows species specific results: (A–E) *A. alburnus*, (F–J) *C. gobio*, (K–O) *L. lota*, (P–T) *S. lucioperca* and (U–Y) *S. glanis*. The first column is the consensus model of current species distribution based on all repetitions of GAM, GLM and RF, the second shows the result of the ensemble forecasting based on the combination of all SDM's (including repetitions) and all climate projections, followed by the maps showing the mean consensus model based on all SDM's (including repetitions) and ECHAM5, HADCM3 and IPSL-CM4 climate projections, respectively.

Estimation of the future distribution patterns was performed for species from group (3), because for these species climate was more important than for all other species groups. From group (4), whose species are affected in general by all three studied factors (climatic, hydromorphologic and topographic), future distribution of the stenothermal L. lota was also assessed. Figure 4 shows potential future habitat suitability maps for C. gobio for different statistical approaches and different climate models. The apparent decrease in the habitat suitability is congruent with predicted temperature increase for all climate models (see Figure 3 (e–h) and Figure 4). However, Figure 4 and Figure 5 indicate significant variability for different statistical approaches as well as for the different climate models. In terms of the occurrence frequency, future projections for C. gobio vary from 0.10 (GAM & HadCM3) over 0.24 (consensus model merging all projections) to 0.47 (GLM & ECHAM5).The previous imply changes from a tremendous drop to a slight increase compared to the current occurrence frequency (0.42), and consequently underline the high uncertainty of the future projections. In terms of the coincidence rates, the situation is much more positive. Average coincidence rates and the corresponding standard deviations between the GAM and GLM, GAM and RF and GLM and RF projections for species from group (3) were 92±7%, 88±6% and 87±6%, respectively. Differences in the projections of HadCM3 and IPSL-CM4 were rather negligible (average coincidence rate of 97±2%) whereas ECHAM5 projects slightly different patterns then the previous two models with lowest coincidence rate with the HadCM3 model (89±6%). Overall, differences among climate models were much less than differences among statistical methods.

Comparisons between the consensus models for the current and future situation calibrated using species specific predictor variables selected from the “basic set of predictors” indicated that the coldwater adapted species L. lota and C. gobio are expected to lose 45% and 71%, respectively of currently suitable cells (Table 7). The warm water adapted species A. alburnus, S. lucioperca and S. glanis are expected to significantly expand their distribution ranges. Including two additional factors in the model building process (PSeason and Tmax) for the coldwater adapted C. gobio resulted in a significant change of predicted distribution pattern from losing about 45% of the currently suitable cells to gaining about 29%. Thereby, RF estimates were considerably unproportional to that based on GAM and GLM.

**Table 7:** Summary of the mean expected loss and gain.

	GAM		GLM		RF		ECHAM5		HADCM3		IPSL-CM4		Mean	
Code	loss	gain	loss	gain	loss	gain	loss	gain	loss	gain	loss	gain	loss	gain
*Alburnus*	0	160	0	159	9	125	0	142	0	161	0	160	0	158
Alburnus*	0	140	0	161	13	53	0	122	0	141	0	157	0	145
*Cottobio*	67	0	25	2	46	4	32	3	61	0	50	0	45	0
Cottobio*	13	6	5	15	0	109	1	45	3	22	6	8	0	29
*Lotalota*	60	0	77	0	65	9	80	0	60	0	66	0	71	0
Lotalota*	16	23	67	0	4	132	42	5	15	18	14	20	21	8
*Sanderca*	0	166	0	157	0	169	0	147	0	170	0	166	0	166
Sanderca*	0	137	0	159	0	165	0	138	7	168	0	163	0	162
*Siluanis*	0	261	0	269	2	257	0	208	0	283	0	275	0	269
Siluanis*	0	227	0	277	2	220	0	178	11	276	0	267	0	262

Table values indicate mean expected loss and gain (percentage of suitable cells) as compared to the consensus model for the 20th century climate. Asterisk in the species code (*) indicate that the predictor data set was extended by Tmax and PSeason. Model specific values (GAM, GLM and RF) are based on averaging across all future projections of the three climate models. Climate model specific values (ECHAM5, HADCM3 and IPSL-CM4) are based on averaging across all corresponding projections of the three SDMs. “Mean” indicates loss and gain resulting from comparison between the consensus future projection merging all SDMs and all climate change models and the consensus model for the 20th century climate.

Analysis of the current ranges of the model predictors and those predicted for 2050s revealed that AnnTMean is outside the calibration range for 82% of the study area (Table 8). For all other factors, the mismatch between the calibration and the future predictor range was lower than 2%. However, due to the multivariate model setup, on average, for more than 90% of the study area future estimates were based on at least one extrapolated species environmental responses.

**Table 8:** Uncertainty estimation of the future species distributions.

scenario	AnnTMean	Isotherm	TSeason	AnnPMean	Tmax	PSeason	total
ECHAM5	74.0	0.1	3.6	0.3	50.0	0.0	84.0
HADCM3	85.6	0.1	0.4	0.6	83.1	0.0	96.3
IPSL	85.1	0.1	0.1	0.4	63.4	0.0	90.5
mean	81.6	0.1	1.4	0.4	65.5	0.0	90.3

Table values indicate percentage of cells with projected values outside the species calibration range (either lower than observed minimum or higher than the observed maximum). The column “total” is the total number of cells where for at least one model parameter the projected values are outside the species calibration range.

## Discussion

### Methodological aspects

Overall, our study showed a high potential for the use of grid cell related information in biogeographical modeling of fish distribution patterns.

ME has shown to be excellent for modeling distribution patterns of species with low occurrence frequency, however, due to performance dependence on occurrence frequency it is considered inappropriate for biogeographical modeling at the particular scale resolution. Although RF was clearly outperforming GAM and GLM in the model calibration phase, all three methods had similar performance in the validation phase indicating that for predictive purposes, they are all equally suitable. However, since GAM and GLM are both additive species distribution models, when used for conservation and restoration planning, the projections require a thorough revision. Due to the additive consideration of the contributing factors, one could calculate a high probability of species occurrence in a region where all factors except one are within the species environmental tolerance range. However, if this one factor points to the water temperature above species ultimate lethal limit, then it is obvious that the model will fail to give a reasonable estimate of the habitat suitability. A simple solution to this problem is adding an additional algorithm in the probability estimation, which reduces the species occurrence probability or the habitat suitability to zero for all locations where the species existential tolerance limits are exceeded for at least one factor.

The importance of using consensus methods among different SDMs towards assessment of the model inherent uncertainty is already addressed in recent studies (e.g. [1], [6], [7]). However, consensus methodology neither mitigates projection uncertainty due to a weak predictor selection nor projection uncertainty due to poor performing SDMs. To assure reliability of our future projections, explicit parameter selection was supplemented with application of the consensus methodology for only those species with “good” to “excellent” average validation ROC score. The need for a well-grounded predictor selection process and detailed model validation was confirmed by the fact that RF provided “excellent” models for all calibration samples independent of the predictor data set used as well as with a significant change in the estimated future “losses” and “gains” following a change in the predictor setup. Namely, with including two more parameters in the modeling, due to low to moderate predictive power of the initially considered predictors, predictor hierarchical importance was significantly rearranged followed with changes in their contribution to the probability of occurrence. Consequently, estimates of climate change effects were significantly different from those of the initial model.

Uncertainties associated with the estimates of the climate change effects on fish distribution patterns across the TK25 grid cells are high and have multiple sources. Consistent with the results of Buisson et al. [6] we have shown that SDMs contribute to higher variability in future projections than the climate change models. Since most ecological studies on climate change effects were based on the combination of GAM with the HadCM3 model we outline that our results show that this particular combination led to the most pessimistic estimates of future habitat suitability, whereas the most optimistic one was the combination of GLM and ECHAM5 model.

As the most concerning uncertainty source we identified a mismatch between the current and future ranges of factors affecting species distributions. On average, for more than 90% of the studied area at least one factor is expected to be beyond the range considered in the model calibration, implying that this effect has to be necessarily included in the process of evaluation of the projection uncertainty, independently of the study grain (site, catchment or grid cells). We outline that in order to obtain reliable estimates of future changes, not only parameter-, SDM- and climate model selection should be performed with care but also selection of the study area. Extending the study area to regions with high overlap of current environmental conditions with the future expected conditions in the area of interest is a potential solution for avoiding the above described pitfall.

### Species' environmental responses

Despite the uniqueness of our scale resolution, our results with respect to the type of climatic and topographic factors affecting fish species distributions were in accordance with recently observed general trends (see [2], [3], [9]). For instance, using GAM models calibrated on site data for 30 fish species from major French rivers Buisson et al. [2] have shown that the upstream-downstream gradient accounts for the major portion of the variability in species niche separation. The previous comply with our results as well as the conclusion that the mean annual temperature should be considered for both cold and warm water species. Our analyses indicate that neither human population density nor land use have considerable predictive power with respect to the distribution patterns of the analyzed fish species. The fact that Germany has low population density variations as compared with other European countries might be the reason for the low predictive power of population density. Regarding land use, we assume that the size of our analysis unit was on one hand too small to catch the accumulated effects of the land use in the basin (e.g. agricultural land use and consequent nutrient loads) and on the other hand, too coarse to catch the local effects (e.g. shadow effects caused by trees). The necessity of describing basin heterogeneity when describing fish species distribution patterns is confirmed by the predictive power and the importance of the cumulated upstream river length and the Strahler order. The cumulated upstream river length describes the general biodiversity potential whereas the Strahler order describes the complexity level of the stream, and, consequently, the habitat heterogeneity level regarding the flow regime. Strahler order provided a suitable proxy for the upstream-downstream gradient and river type by integrating over all hydro-physical characters along the river network. Our result that the Strahler order alone describes a large portion of spatial distribution pattern of *B. barbus* is not surprising considering that this species generally occupy middle stream reaches. Also, significant univariate ROC score and high predictive importance for the factor describing dominant substrate type underlined the importance of this factor in biogeographical modeling of fish species and confirmed substrate preference as an important fish trait [21].

Through analysis of the species' environmental responses we have shown that *P. pungitus*, *C. taenia* and *L. fluviatilis* occupy similar environmental niche well described by the TK25 cell altitude. Actually, all of these species tend to occupy stream reaches close to the river mouth [33]. Lassale et al. [4] have shown that the spatial distribution pattern of *L. fluviatilis* across Europe is mainly dependent on the longitude at the mouth. Considering that Germany has a pronounced North-South altitudinal gradient, this is comparable to our finding that the average altitude of the studied region is the most important environmental predictor. *Salmo trutta*, *P. phoxinus* and *T. thymallus* were found strongly related with the hydromorphologic factors. They all require clean gravel substrates [34] and tend to occupy the upstream basin parts characterized by fisheries as “trout zone”. *S. lucioperca*, *S. glanis*, *A. alburnus* and *C. gobio* showed to be driven by climatic and hydromorphological factors occurring in stream middle reaches, but the first three tend to occupy deeper, low flowing waters in large streams, while *C. gobio* is restricted to fast-flowing water of small stream to medium-sized rivers [33]. Within the analyses of the potential of climate as a single factor describing fish species distribution both, *C. gobio* and *S. glanis* have been identified as particularly climate sensitive wherefore the finding on the importance of hydromorphology for these two species adds significant information with regard to the potential conservation pathways.

### Expected changes in fish distribution patterns with climate change

Climate change is expected to cause alterations in both temperature and precipitation in Germany. We have shown that the climate alterations are likely to affect fish distribution ranges, manifested as narrowing of ranges of cold water fish (*C. gobio* and *L. lota*) and expansions for warm water species; however, the extent of these changes is highly uncertain.

Analogous to marine fish populations (e.g. [35]) we expect that as an implication of the climate change effects, freshwater organisms will face complex changes at organismal-, individual-, population and ecosystem levels of biological organisation, which will vary in magnitude and area among the different regions. Individual-level changes will involve movement into more suitable areas and consequently change species composition along the upstream-midstream-downstream gradient. Such a tendency has already been observed for the Upper Rhône River [36] where the increase of average water temperature in the last quarter of the 20th century by about 1.5° was followed by the replacement of cold water, northern species by thermophilic, southern species. Population level changes might be caused by local climatic changes. For example, Borgstrøm et al. [37] have shown that little or no snow, accumulated snow depth and summer temperatures have considerable effects on the population recruitment of brown trout in high mountain areas of Norway. Further, physiological and ecologic changes in populations of Atlantic salmon and brown trout involving time of spawning, egg survival rate, longevity and age and size at smelting are strongly affected by temperature [38], [39]. At the ecosystem level it is expected that the strength of trophic interactions between consumers and resources may become weakened or broken [15]. Also, increased temperatures have shown to lead to rejuvenation and increase of small fish abundance [40], [41], implying that higher temperatures might affect processes related to nutrient cycling. The importance of fish in nutrient cycling may be most pronounced during dry periods when external inputs are reduced [42], and drier periods are predicted to be more frequent, or of longer duration in many areas (ref to climate model). Woodward et al. [15] also noted that summer drought may lead to general habitat degradation through increase of pollutant concentrations. In addition, ecosystem changes will vary among ecosystem types. For example, small streams, due to greater temperature and flow sensitivity, are expected to be more vulnerable than large streams to the future climate changes.

Secondary effects of the individual level changes, manifested as species establishments outside their current native spatial ranges and the subsequent invasions, might be even higher than direct temperature effects. For example, for common carp, currently non-native persistent but not established in England and Wales, it has been shown that the combination of temperature increase and carp invasion will result in habitat destruction, macrophyte loss and increased water turbidity [43]. Expansion of invasive species will modify biotic interactions by altering competitive dominance, increased predation rates, as well as enhancement of disease virulence [44].

### Study limitations and recommendations

Despite high predictive performance of the SDMs based on grid cell related species information, we underline that the grid cell data should be seen only as an alternative in cases where stream network related data are not available. Especially in regions where the major factors affecting species distributions are dispersal limitations and local environmental peculiarities acting at spatial scales much smaller than the grid cell size, the stream network related data (site or river reach) have the primacy over the grid cell data. One of the major limitations of SDMs in general are the assumptions that species are in equilibrium with their environment and that there is no limitation to dispersal (see [45]). Fish species generally require different habitat properties for different life stages implying that a critical criterion for completing their life cycles is accessibility of functionally required habitats along the river systems [12]. For example, dams can block access to the historical spawning places and also induce habitat changes such as retention of nutrients and sediments, and can even lead to the genetic fragmentation of populations [46]. Also, possible adaptive shifts in environmental tolerances, especially when the species ability to disperse or migrate is limited, are ignored by the common SDMs [10]. However, even if all of the above listed SDM limitations have been carefully taken into account, the spatial study extent that covers only a fraction of species' realized niches [47] or a fraction of possible environmental characteristics of freshwater systems would seriously affect the prediction reliability of the effects of future ecosystem changes.

The previous considerations imply that, in order to account for the whole spectrum of climate change effects on fish populations, a multi-faceted approach is needed, including appropriate long term field data at a sufficiently broad spatial scale, information from experimental studies designed to understand species' environmental responses, establishment of a robust analysis framework and thorough evaluation of the uncertainties associated with estimates. Only with these major steps, will we achieve confidence in the ability of the methodology to model the current and predict the future effect of environmental changes.

## Supporting Information

Figure S1 **Predictor maps:** annual mean temperature (AnnTMean), mean diurnal temperature range (DiuTRange), isothermality (Isotherm), mean temperature seasonality (TSeason), maximum temperature of warmest month (Tmax), mean temperature of wettest quarter (TWetQuar), mean temperature of driest quarter (TDryQuar), annual mean precipitation (AnnPMean), mean precipitation seasonality (PSeason), mean altitude (AltMean), maximum cumulative length of the upstream flow network (CumLenkm), maximum Strahler order (Strahler), dominant river type (RtypMost), dominant land use type (Lusemax) and mean population density (Popmean).
(TIF)
