# Supplementary figures and images for: Where Are All the Fish: Potential of Biogeographical Maps to Project Current and Future Distribution Patterns of Freshwater Species
Source: PLoS One. 2012 Jul 6;7(7):e40530. doi: 10.1371/journal.pone.0040530 (PMC3391242; doi:10.1371/journal.pone.0040530)

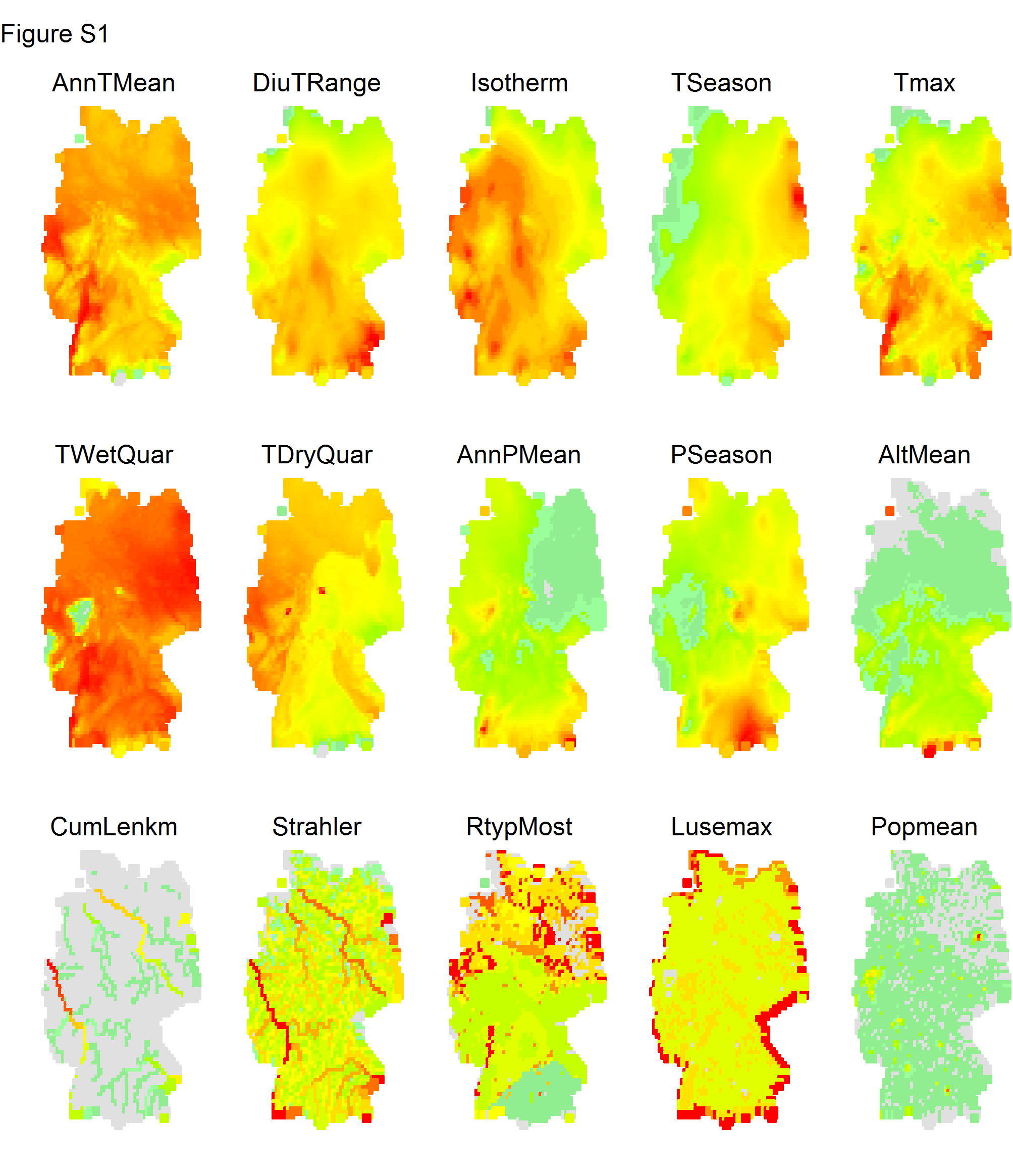

Supplement: Figure S1 — Predictor maps: annual mean temperature (AnnTMean), mean diurnal temperature range (DiuTRange), isothermality (Isotherm), mean temperature seasonality (TSeason), maximum temperature of warmest month (Tmax), mean temperature of wettest quarter (TWetQuar), mean temperature of driest quarter (TDryQuar), annual mean precipitation (AnnPMean), mean precipitation seasonality (PSeason), mean altitude (AltMean), maximum cumulative length of the upstream flow network (CumLenkm), maximum Strahler order (Strahler), dominant river type (RtypMost), dominant land use type (Lusemax) and mean population density (Popmean). (TIF) [file pone.0040530.s001.tif]
